# Investigation of the Formation of Squalene Oligomers Exposed to Ultraviolet Light and Changes in the Exposed Squalene as a Potential Skin Model

**DOI:** 10.3390/molecules27113481

**Published:** 2022-05-28

**Authors:** Matteo Zecchini, Robert A. Lucas, Cameron Robertson, Tomris Coban, Ravtej Thatti, Adam Le Gresley

**Affiliations:** 1 Life Sciences, Pharmacy and Chemistry, SEC Faculty, Kingston University, Kingston-upon-Thames KT1 2EE, UK; zecchini.matteo@libero.it (M.Z.); c.robertson@kingston.ac.uk (C.R.); t.coban@kingston.ac.uk (T.C.); r.thatti@kingston.ac.uk (R.T.); 2 GlaxoSmithKline Consumer Healthcare, Weybridge KT13 0DE, UK; robert.a.lucas@gsk.com

**Keywords:** squalene, oligomerisation, NMR, DOSY

## Abstract

UV-induced oligomerisation of squalene was undertaken to indicate the potential for squalene-containing biological systems to exhibit rheology changes. DOSY NMR enabled the determination of the molecular weight (MW) range using Stokes–Einstein Gierer–Wirtz Estimation (SEGWE Calculator, University of Manchester). This approach was validated by Atmospheric Solids Analysis Probe Time of Flight Mass Spectrometry (ASAP TOF MS). To demonstrate the principle, both benzoyl peroxide and AIBN were used, separately, to initiate rapid, radical oligomerisation. Subsequent experiments in the absence of initiators compared the influence of UV wavelength and time on the resulting oligomer formation. To further model a relevant biological implication of this potentially chaotic UV oligomerisation, both saturated and unsaturated free fatty acids were added to squalene and exposed to UV at 285 nm and 300 nm to determine if cross oligomerisation could be observed. This representation of sebum evidenced the formation of a distribution of higher MW oligomers. Internal viscosity was normalised using the DMSO solvent, but to confirm that changes in rheology did not affect diffusion, a final experiment where fresh squalene was added to our oligomer mixture, representative of sebum, showed that unchanged squalene possessed the anticipated monomeric diffusion coefficient and hence MW. This work suggests, at least qualitatively, that UV-induced squalene oligomerisation can occur over time and that this may have a role in the behaviour of squalene on the skin.

## 1. Introduction

Of all the skin layers, the *Stratum corneum* and the skin surface lipids (SSL) are routinely exposed to the highest level of UV radiation. Previous studies have demonstrated that UVA exposure increases the formation of reactive oxygen species (ROS) in the skin’s upper layers, SSL, which cover the *Stratum corneum* in the sebaceous gland-rich regions [1,2,3]. Studies have shown that squalene monoperoxide formation in human skin is strongly induced by UVA radiation to a greater extent than UVB radiation. The same study has shown that peroxidation induces the formation of a mixture of squalene peroxide isomers (up to six) that have been characterized by NMR, although only partially [4].

Squalene peroxides have also been demonstrated to contribute more than other peroxides to comedogenesis. Ottaviani et al. investigated the possible role of squalene peroxide as the triggering factor in the development of the inflammatory process of using a human keratinocyte cell line by increasing lipoxidase (LOX) activity. The same study concluded that squalene peroxide can induce an inflammatory response in the cell line under investigation through LOX activity and increases the expression and secretion of the proinflammatory cytokine IL-6 [5]. Sebum has been subject to numerous publications, but there appears to be a lack of agreement regarding its composition [5,6]. The main constituent of the sebaceous lipids in sebum have been identified as squalene (the single most abundant component ≈15%), triglycerides (TG), free fatty acids (FFAs), wax esters, cholesterol (CHOL) and cholesteryl esters. The other constituent of sebum is the extracellular lipids, which are made mostly of ceramides, FFAs and CHOL [1,2].


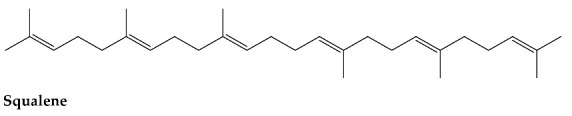


This study focuses on squalene and its structural changes after being exposed to ultraviolet radiation (UVR), which has been shown to be the major source of cutaneous oxidative stress that can be related to skin ageing and skin cancer. Specifically, we investigated how the radical and photochemically catalysed oligomerisation of squalene could occur in nature, resulting in the increase in viscosity observed within comedones. In order to demonstrate this as a possibility, a combination of MS and NMR techniques were used to evidence this oligomerisation of squalene when exposed to light with wavelengths of 285 nm and 300 nm in the presence and absence of radical initiators over a period of several days. The rationale behind this was to mimic UVB/UVA resp. exposure at the two wavelengths and to simulate squalene in the sebum exposure over a period of days of that exposure to better reflect the real-world exposure of skin in the summer. It was also decided that to better mimic sebum, saturated and unsaturated free fatty acids were added to determine if cross oligomerisation could be observed. The time periods chosen (days rather than hours) are better representative of the exposure of squalene in sebum to sunlight during the process of comedone formation.

NMR was chosen as a main analytical technique owing to the high dimensionality that affords characterisation as well as quantitation. The use of DOSY as a powerful technique to infer molecular weight from the diffusion parameter has now gained wide acceptance as a method to track the reactions of this nature in situ [7]. In addition, ASAP MS was undertaken to evidence not only the formation of higher molecular weight species in general, but also to demonstrate why MS does not afford the granularity of analysis when dealing with mixtures of non-polar molecules whose fragmentation is often via the sequential cleavage of CH_2_ groups.

## 2. Results

### 2.1. ^1^H and DOSY NMR

**Figure 1:**
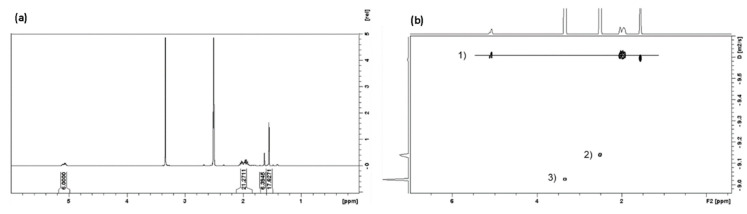
Proton NMR of a commercially available squalene sample (**a**) and DOSY NMR of the same sample (**b**). (1) squalene, (2) DMSO residual and (3) H_2_O. Both spectra were recorded in DMSO-d_6_.

Squalene was analysed by ^1^H and DOSY NMR and the spectra recorded are presented in Figure 1 below and reflect the situation at *t* = 0, before any UVR was applied to the sample. The DOSY NMR recorded for squalene shows the diffusion coefficient can be unequivocally assigned for all the peaks belonging to squalene. It is worth noting that both radical initiator and photochemically-induced changes to the squalene resulted in a yellow oil of greater viscosity than squalene being generated. This is accounted for using the internal viscosity standard to correct for this variation as specified in the method.

### 2.2. Irradiation of Squalene

The effect of a radical initiator on inducing the radical oligomerisation of squalene was evaluated using Benzoyl peroxide and AIBN and exposing them for 5 ½ days under a 285 nm UV light. Data in Supporting Information (Section S1) indicate the changes provoked by the UV light in terms of alkene peak broadness and also the appearance of a very broad multiplet in the aliphatic region around 0.8–1 ppm, which is usually the region where non-allylic CH_2_/CH_3_ groups are found. From the DOSY NMR, these new broad signals are indicative of species of a higher MW. It is also evident that there appears to be a limited difference in the result for the two different initiators.

**Figure 2:**
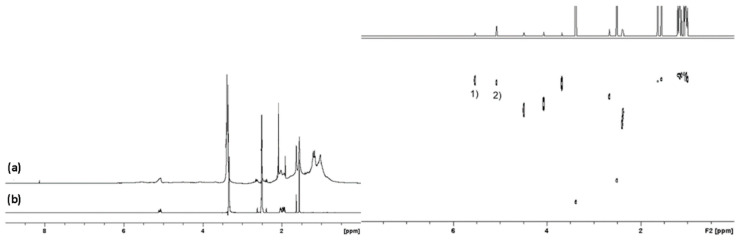
(**Left**) ^1^H-NMR comparison of (a) squalene under 285 nm UV light for 10 days and (b) squalene not exposed to UV light. (**Right**) ^1^H DOSY NMR spectrum showing the diffusion signals (1) and (2) for the putative oligomeric squalene.

Squalene alone was subsequently irradiated under 285 nm (10 days) and 300 nm (7 and 10 days) UVR. The representative spectrum in Figure 2 shows similar changes to those observed in the initiator catalysed reaction, i.e., peak broadening and also the appearance of a very broad multiplet in the aliphatic region around 0.8–1 ppm (See Supporting Information S2 and S3). This suggests that the reaction that occurs photochemically potentially involves the generation of allylic radicals. This is as opposed to a concerted [2 + 2] Diels–Alder type reaction.

**Figure 3:**
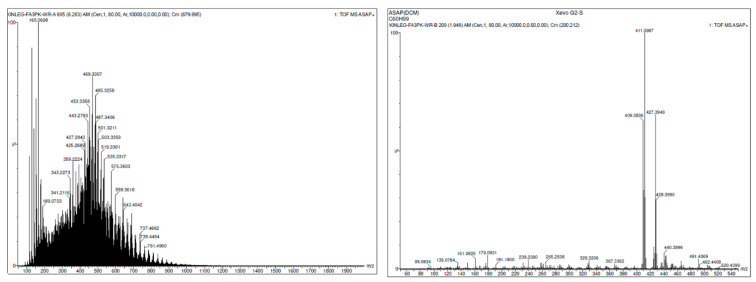
(**Left**) ASAP TOF Mass Spectrometry analysis of a mixture of unsaturated hydrocarbons with a wide range of molecular masses observed from *m/z* 150 to at least *m/z* 1000 according to the National MS centre, EPSRC, Swansea (**Right**) ASAP TOF Mass Spectrometry analysis of non-irradiated squalene.

In an effort to validate the NMR observations, the 285 nm 10 day squalene sample was analysed using TOF MS with an Atmospheric Solids Analysis Probe (ASAP) (Figure 3). For comparison, a neat sample of non-irradiated squalene showed the expected molecular ion at *m/z* = 411. The MS spectrum for the irradiated squalene broadly supports the diffusion-derived MW data; that is to say, the squalene undergoes a conversion into a potential mixture of species of higher MW. This is further supported by the complex fragmentation pattern that is often seen with CH_2_ rich oligomeric systems, which argues against the formation of squalene oxides, which are equally not observed in the NMR (2,3-oxidosqualene CH-O comes at 2.6 ppm).

### 2.3. Sebum Mimicking Squalene Preparation

#### Irradiation at 285 nm and 300 nm

**Figure 4:**
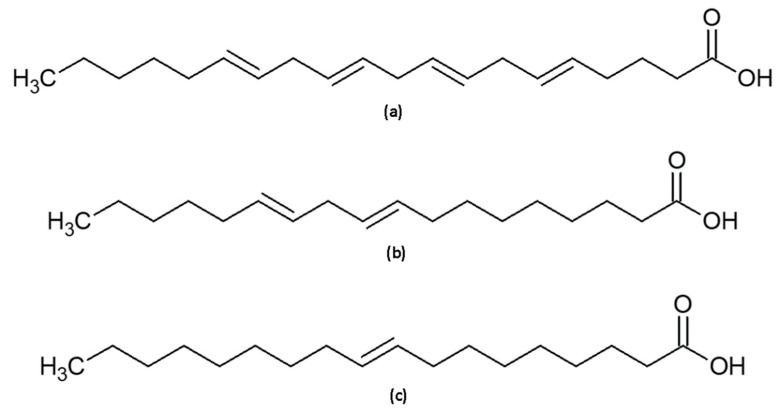
Chemical structure of the FFAs in the mixture: (**a**) arachidonic acid, (**b**) linoleic acid and (**c**) oleic acid.

**Figure 5:**
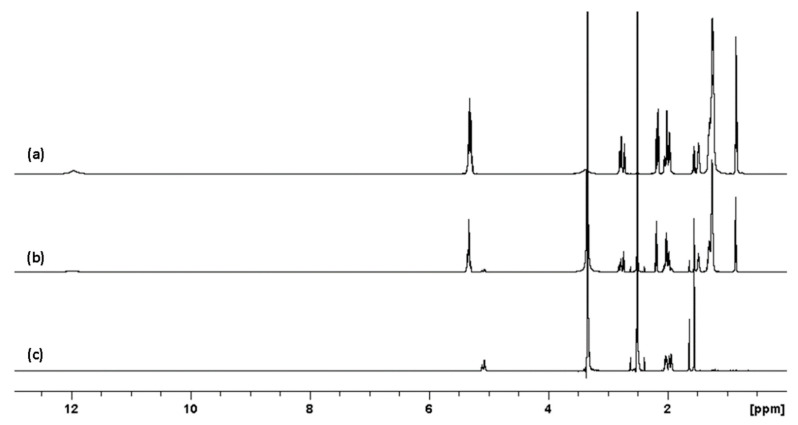
Proton NMR overlap for FFAs mixture (**a**), FFAs and squalene mixture (**b**) and squalene alone (**c**) in DMSO-d6. From Figure 5 above, it is evident that the unsaturation of the squalene and fatty acids resonates at slightly different chemical shifts, enabling potential identification of which substrates may be polymerising. Some of the proton signals of the FFAs (2.7–2.6 ppm) can also indicate crosslinking formation as the vinyl protons should shift as a result of radical addition to double bonds.

To evaluate the possibility of crosslinking between squalene and FFAs under UV light radiation and, hence, mimic a more typical heterogenous biological environment, preparations of oleic, linoleic and arachidonic acids mixed with squalene were subjected to UVR (285 and 300 nm) over several days. The chemical structures of the FFAs are shown in Figure 4 along with the typical ^1^H spectra for these FFAs prior to exposure to UVR (Figure 5).

**Figure 6:**
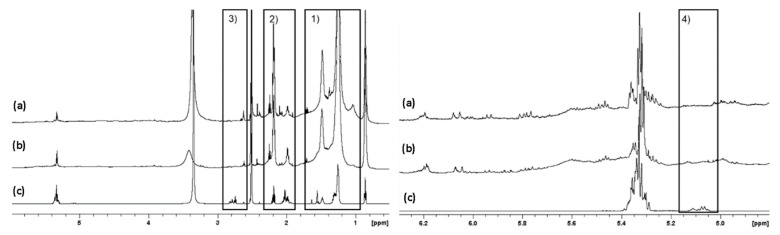
(**Left**) ^1^H-NMR of residue exposed to (a) 300 nm for 12 days, (b) 285 nm for 10 days and (c) starting mixture not exposed to UVR (c), where differences can be seen due to broadening (1) change in ratio (2) and peaks disappearance (3). (**Right**) An enlargement of the chemical shift region 6.3–4.8 ppm shows the squalene unsaturation (4) for exposure to (a) 300 nm for 12 days, (b) 285 nm for 10 days and (c) starting mixture not exposed to UVR.

The enhanced complexity of the ^1^H NMR spectra can be evidenced in Figure 6, where the loss of vinylic protons, coupled with the growth of non-allylic CH_2_/CH_3_ signals, point towards this complex oligomerisation that could involve FFAs as well as squalene. The complex distribution of the components formed in this chaotic arrangement of reactions is evidenced in the Supporting Information (Section S4).

### 2.4. Verification of Reaction as Opposed to Rheology

To evidence that the signals indicating unsaturation, appearing in the ^1^H-NMR after squalene was exposed to UVR, were genuine “new” signals, and that the MW extrapolated from DOSY NMR data was not the result of a change in viscosity, squalene was spiked into an aliquot taken after seven days of UVR exposure (285 nm) and analysed by both Proton and DOSY NMR (Supporting Information S5). In the spiked sample, the squalene was shown to diffuse as expected and did not overlap the new signals associated with the new components on the DOSY.

To further evidence that the reaction of squalene can be photochemically driven, a squalene sample was exposed to ambient laboratory light over a period of 54 days. The NMR spectra (Supporting Information Sections S6 and S7) indicate the sample remains largely unchanged; however, within the range of 5–11 days new upfield signals (as opposed to those observed downfield when exposed to UVR) are seen that potentially suggest some reversible cyclic species, which appear to be in equilibrium with squalene as the new signals appear to be much sharper than those observed in the photochemically-driven experiments and revert back to squalene over time. It is also clear from the NMR of squalene upon ambient exposure that the generation of large and broad non-allylic CH_2_/CH_3_ signals at 1.2 ppm do not occur.

## 3. Discussion

**Figure 7:**
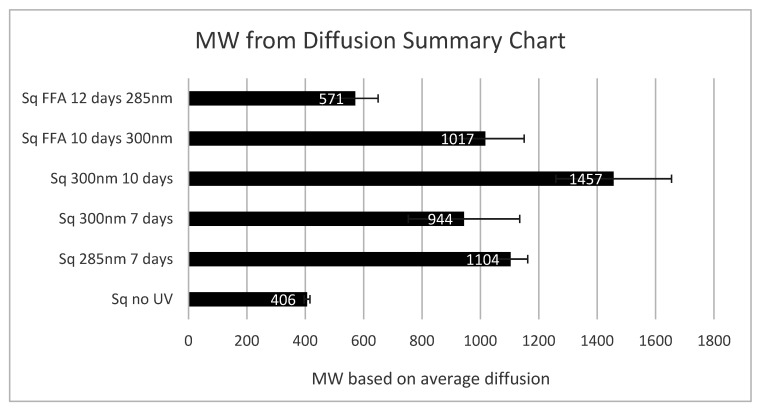
Average MW as determined by DOSY NMR for signals observed at 5.07 ppm and 5.54 ppm (correlating to vinylic protons) for squalene (Sq) and a mixture of squalene and linoleic/oleic and arachidonic acids (FFA), irradiated over a specified number of days at 285 or 300 nm. Error bars show standard deviation of MW.

Whilst the presence of six double bonds on a single molecule exposed in a non-targeted fashion to UVR or radical initiation clearly leads to a complex and chaotic series of inter and intramolecular reactions, the authors are unable to clearly identify one specific species. Indeed, the combination of ^1^H NMR, DOSY and ASAP MS evidences a mixture of compounds that result from the addition of one or more double bonds by squalene reacting with both itself and with the FFAs (Section 2.2) in the presence of UVR. The MW comparison derived from the DOSY analysis of the average vinylic signals shows a range of oligomeric species present after the exposure of squalene to UVR over several days. The extrapolated MW data shown in Figure 7 indicate that trimeric squalene oligomers may indeed form and that the presence of FFAs either retards this process or it undergoes a reaction with the squalene to reduce the average observed molecular weight for the species observed within the vinylic region of the NMR. This formation of large fatty acid oligomers is observed in nature [8,9].

This study attempts to model the effects of UVR on squalene over a relevant timeframe and using similar intensities to those that the skin may encounter during the summer months. In stressing the limitations of this study, however, it is important to note that skin and comedone biochemistry is complex, with many high MW triacylglycerides (TAGs), diacylglycerides (DAGs) and ceramides forming key parts of this structure. It is owing to the complexity of the TAG/DAG/ceramide mixtures and their otherwise intractable nature that this controlled oligomerisation was undertaken. It is important to note that for the analysis of tissue samples to detect such oligomers in skin, considerable purification and concentration would be required to enable NMR to be a useful technique. Even 3D MS techniques such as desorption electrospray ionization (DESI) imaging MS would have to discriminate between a wealth of lipids with similar fragmentation patterns.

Whilst further work should be undertaken to evidence the actual natural formation of squalene oligomers in biologically relevant samples, the proof of principle exists, as evidenced by the analysis undertaken here, that the reaction (most likely radical driven) of squalene with itself shows both higher MW species being formed as well as the growth of non-allylic CH_2_/CH_3_ groups. This is compelling evidence that double bonds are being lost during this photochemically-driven process and that chaotic oligomerisation is taking place.

## 4. Material and Methods

### 4.1. Materials

All the chemicals involved such as squalene, benzoyl peroxide, AIBN, oleic, linoleic and arachidonic acids were purchased from Sigma-Aldrich with exception of DMSO-d6 purchased from FluoroChem and were used without further purification. DMSO-d6 was used to record all the ^1^H and DOSY NMR of the mixtures obtained after UV exposure. The microscope slides used to deposit the sample were purchased from Sigma-Aldrich.

Single-use 5 mm NMR tubes (Product code 502-7) were purchased from GPE Scientific.

### 4.2. Irradiation Methods

The UV light source was provided by GlaxoSmithKline Consumers Healthcare, Weybridge, UK and consists of a Triple Wavelength PearlBeam (255 nm, 285 nm and 300 nm) by Aquisense Technologies (Erlanger, MA, USA) and equipped with a Hand-Held Light Meter and Optometer (ILT2400) made by International Light Technologies (Peabody, MA, USA), which for our purposes was not necessary.

Methanol, Toluene, Acetonitrile, Ethanol, Chloroform and water (Optima grade) used for MS analysis were purchased from Fisher Scientific (Loughborough, UK). MS analysis using ASAP TOF was undertaken by the EPSRC National Mass Spectrometry Facility in Swansea, UK.

All photochemical experiments involved the addition of squalene preparations onto a glass microscope slide followed by exposure to the PearlBeam with the slide being maintained at 35 °C. In the case of initiator-driven experiments, 50 μL of 50 mM Benzoyl peroxide (in toluene) or 50 μL of 63 mM AIBN (in toluene) were combined with 50 μL of squalene. For the neat squalene experiments, 100 μL was added to the microscope slide, and for the FFA experiments, a mixture off 50 μL of each of the oleic, linoleic and arachidonic acids was mixed with the squalene before 100 μL of the preparation was added to the microscope slide. This use of a thin film on a slide was designed to give maximum surface area for any possible reaction.

### 4.3. NMR Methods

All NMR spectroscopy experiments were conducted on a Bruker Avance III 600 MHz three-channel FT-NMR spectrometer (Coventry, UK), equipped with a TXI ^1^H/2D {^13^C, ^15^N} probehead. The NMR spectrometer is automated using Bruker IconNMR 5.0.7 (Bruker UK Ltd., Coventry, UK), and all spectra processed with Bruker TopSpin 3.5.7 (Bruker UK Ltd., Coventry, UK), as the control software and processing software and Dynamics Center 2.4.9 (Bruker UK Ltd., Coventry, UK), for DOSY analysis. The MW prediction from the DOSY was calculated accordingly to SEGWE calculator made available by the University of Manchester NMR methodology group also in the form of an excel spreadsheet based on a general method extended later and available in the literature [9,10,11,12]. DMSO was used as the internal standard to account for viscosity.

Acquisition of parameters for standard experiments (1D ^1^H and ^1^H DOSY): 1D ^1^H was performed using noesypgpr1d pulse, P1 = 7 μs; flip angle = 90°; D1 = 10 s, NS = 1024. ^1^H DOSY NMR (16 scans) was conducted with a linear pulsed field gradient over 64 steps using the ledbggp2s pulse.

## Data Availability

Data available on request due to restrictions.

## Supplementary Materials

The following are available online at https://www.mdpi.com/article/10.3390/molecules27113481/s1, Supporting Information Sections S1–S7

## Sample Availability

Not available.
