# Supplementary material for: Investigation of the Formation of Squalene Oligomers Exposed to Ultraviolet Light and Changes in the Exposed Squalene as a Potential Skin Model
Source: Molecules. 2022 May 28;27(11):3481. doi: 10.3390/molecules27113481 (PMC9182105; doi:10.3390/molecules27113481)
Supplement: Supplementary file 1 [file molecules-27-03481-s001.zip › molecules-1722166-supplementary.pdf]

Supporting Information

S1 UV exposure of squalene in the presence of initiators

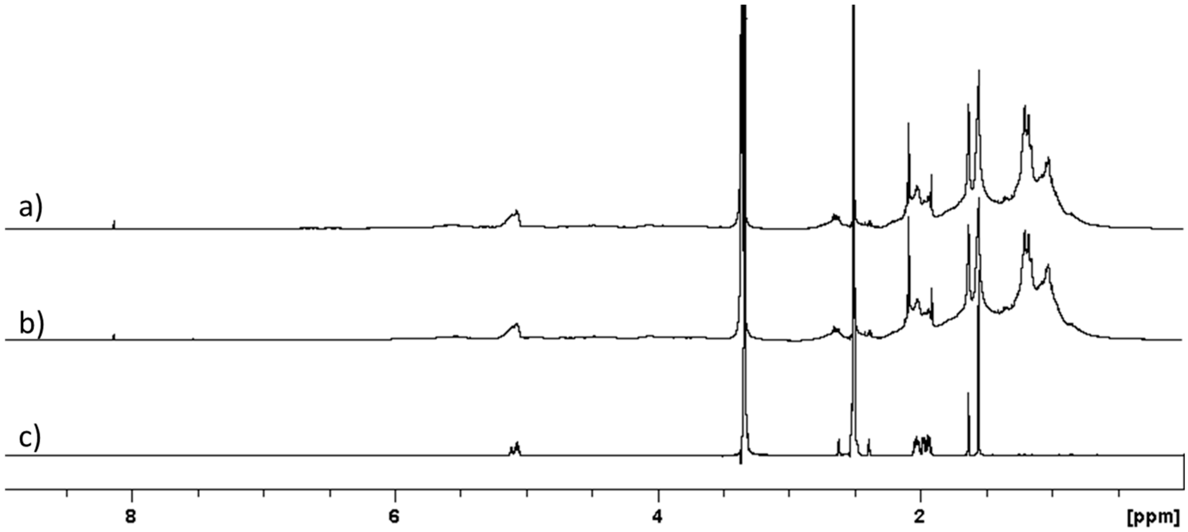

Figure S1. 1H-NMR comparison among a) squalene, AIBN and toluene b) squalene, BP and toluene under 285nm UV light for 5 ½ days and c) a squalene sample in toluene not expose to UV light c).

DOSY NMR analysis of the new signals indicate species of higher MW than the original squalene (Figure S2). evidence that the viscous yellow residue generated is a mixture of partially unsaturated oligomers belonging to a higher MW oligomer rather than squalene itself.

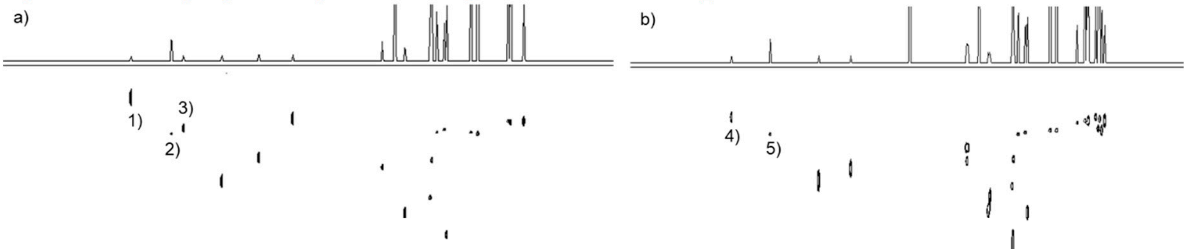

Figure S2. DOSY-NMR for a) squalene & Benzoyl peroxide and b) squalene & AIBN after exposure to UV light
285nm for 5 ½ days.

Table S1. Molecular weight determined from DOSY data for irradiated and non-irradiated squalene in the presence of radical initiators.

Components	Peaks	Diff.Coeff ($x10^{-10}$) m/s^2	Predicted MW
		No exposure	
squalene	5.11ppm	2.43	399
(MW 411)	5.07ppm	2.39	413

		285nm 5 ½ days	
squalene & Benzoyl peroxide	1) 5.52ppm	1.19	1963
	2) 5.07ppm	1.47	1199
	3) 4.93ppm	1.42	1298
squalene & AIBN	4) 5.55ppm	1.24	1781
	5) 5.07ppm	1.40	1341

S2 UVB Irradiation of squalene (285nm)

Diffusion coefficients and calculated MW are presented in table S2 and compared to the same parameter obtained for the squalene starting material. The Diffusion coefficient difference suggests oligomer formation.

Table S2. Diffusion coefficient for irradiated vs non-irradiated squalene over 10 days *prefixed numbers are the signals indicated in Figure 3

UV Irradiation (nm)	Chemical Shift signals	Diff.Coeff ($*10^{-10}$) m/s^2	Predicted MW
None	5.11ppm	2.43	399
None	5.07ppm	2.39	413
285	1) 5.53ppm*	1.50	1145
285	2) 5.07ppm*	1.55	1063

S3 UVB Irradiation of squalene (300nm)

Squalene was exposed for 7 and 10 days to 300nm UV light. The ^1H NMR spectra for these samples is shown in Figure S3

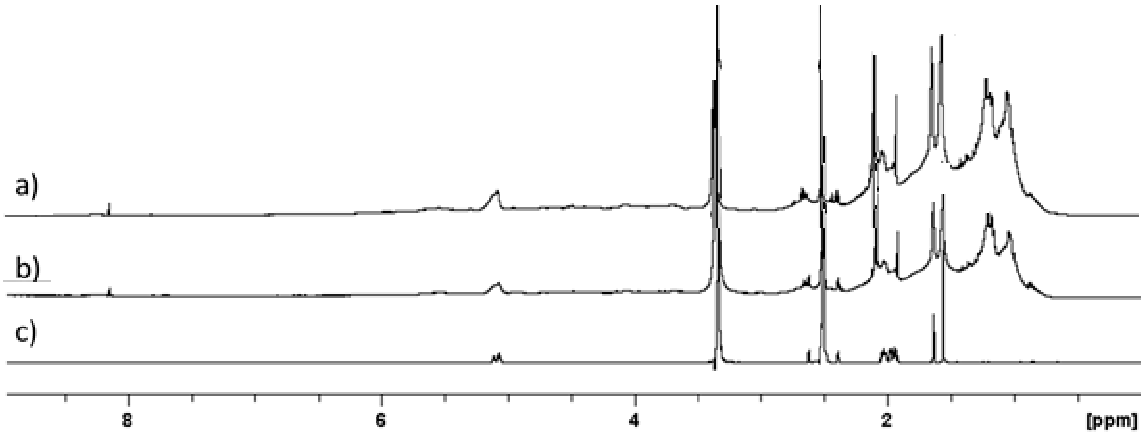

Figure S3. ^1H-NMR comparison among a) squalene 10 days b), squalene 7 days c) squalene sample not exposed to UV light DOSY-NMR shows higher MW oligomers as observed for the shorter wavelength.

The predicted MW and the experimental Diffusion coefficients given are presented in the table below (Table S3 & Figure S4) and compared to the same parameter obtained for the squalene starting material

where again the diffusion coefficients for several of the new signals suggests oligomer formation from squalene starting material.

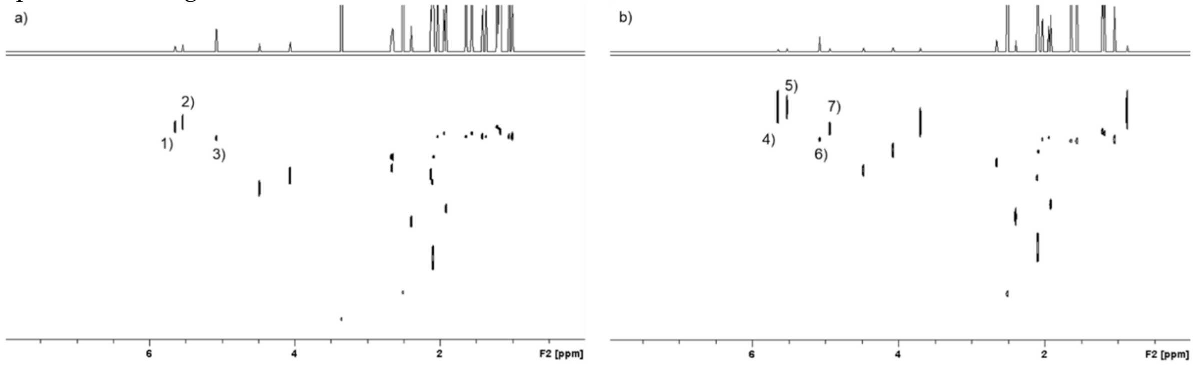

Figure S4. DOSY-NMR for squalene irradiated at 300nm after a) 7days and b) 10 days

Table S3 Diffusion coefficient for squalene irraditated at 300nm over 7 & 10 days compared with non-irradiated squalene. *prefixed numbers are the signals indicated in Figure 6.

UV Irradiation (nm)	Days	Chemical Shift signals	Diff.Coeff (*10^{-10}) m/s^2	Predicted MW
**None**	7	5.11ppm	2.43	399
**None**	7	5.07ppm	2.39	413
**300**	7	1)5.64ppm*	1.59	1003
**300**	7	2)5.54ppm*	1.54	1079
**300**	7	3)5.07ppm*	1.75	809
**300**	10	4)5.54ppm*	1.28	1653
**300**	10	5)5.27ppm*	1.44	1257
**300**	10	6)5.07ppm*	1.35	1460
**300**	10	7)4.94ppm*	1.45	1238

S4 FFA DOSY Diffusion Changes

Changes in the MW where subject to DOSY-NMR analysis reported below (Figure S5).

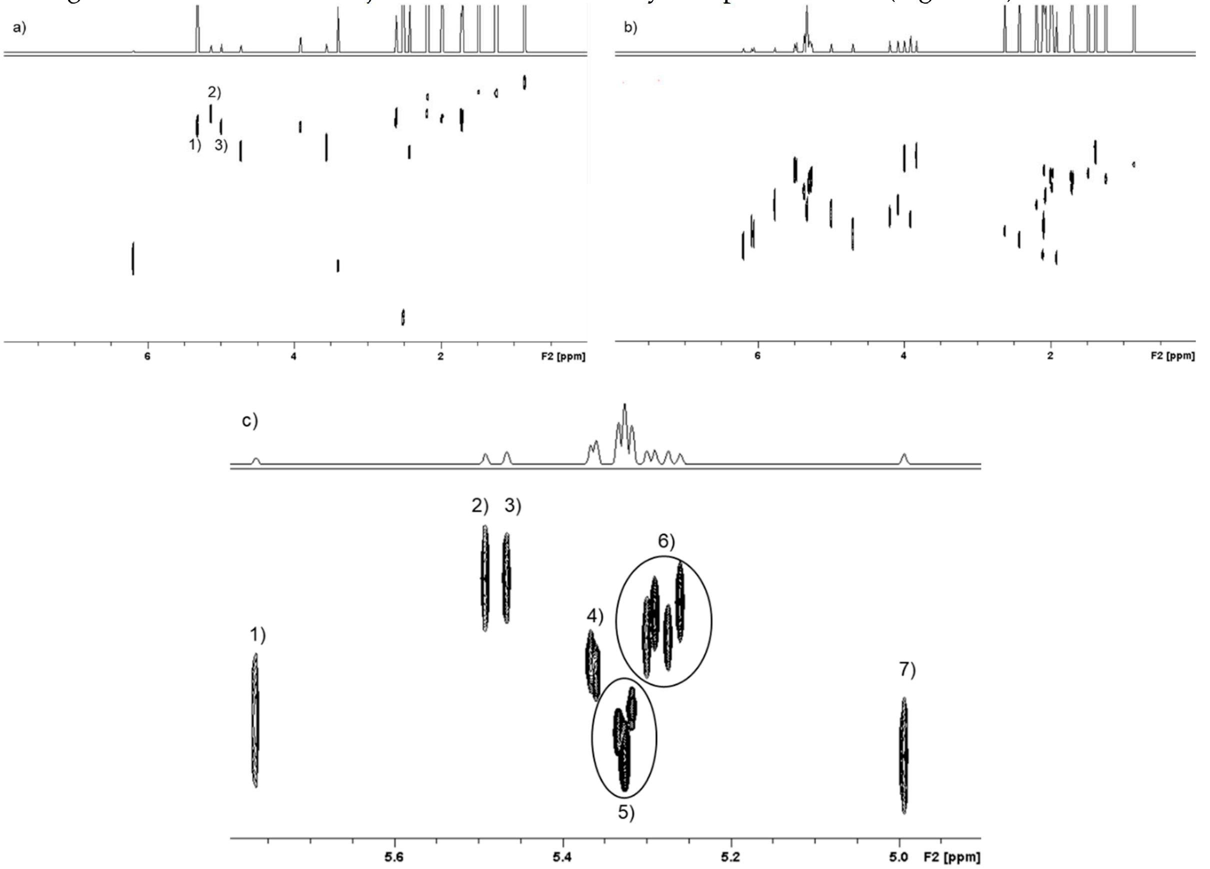

Figure S5. DOSY NMR of residue exposed to 300nm for a) 12days, b) 285nm for 10days and c) enlargement of the b) DOSY spectrum.

Interestingly no peak at 5.06-5.08 was found which would suggest that the squalene may have cross-linked with the fatty acids but nevertheless the MW predicted do not support this conclusion. It's very likely that some sort of cross-linking took place and the remaining unsaturations are too weak/too braod to be correctly detected (Table S4).

Table S4 Diffusion coefficient for squalene/Linoleic/Oleic and Arachidonic acid mixture irraditated at 300/285nm over 10 & 12 days resp. *prefixed numbers are the signals indicated in Figure S5.

UV Irradiation (nm)	Days	Chemical Shift signals	Diff.Coeff (*10^{-10}) m/s^2	Predicted MW
**300**	10	1)5.54ppm*	1.52	1111
**300**	10	2)5.07ppm*	1.65	923
**300**	10	3)4.94ppm*	1.72	841
**285**	12	1)5.76ppm*	2.25	469
**285**	12	2)5.49ppm*	1.94	645
**285**	12	3)5.46ppm*	1.95	638
**285**	12	4)5.37ppm*	2.13	527

**285**	12	5)5.33ppm*	2.27	460
**285**	12	6)5.28ppm*	2.03	585
**285**	12	7)4.99ppm*	2.32	439

S5 squalene spike after 7 days irradiation to account for viscosity change

To evidence that the unsaturation/s appearing on the ^{1}H-NMR after UVR exposure were genuine "new" unsaturation squalene was spiked into an aliquot taken after seven days of UVR exposrure and analysed by both ^{1}H and DOSY-NMR. The ^{1}H NMR before and after the spike are presented below in Figure S6, where is also evident (on the right-enlargement 5.5-0.5ppm) a new set of peaks arise (1, 2, 3).

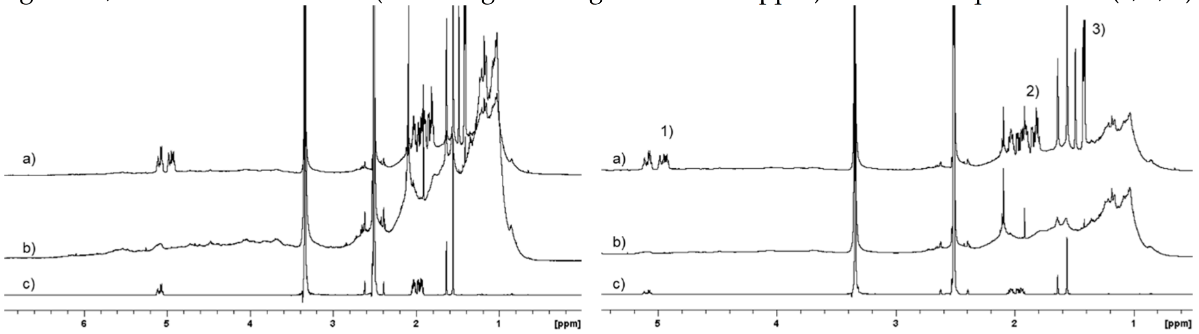

Figure S6. ^{1}H-NMR comparison betweena a) squalene 7days 285nm + Sq-Spike b) squalene7days 285nm and c0 squalene starting material not exposed to UV light.

The DOSY-NMR characterisation (Figure S7 & Table S5) clearly demonstrates that the squalene aliquot resonates clearly at the expected ppm and in table 6 the Diffusion coefficient is not substantially different to the range recorded for the starting material (as in Table 1), which demonstrates that the Diffusion coefficients recorded are not due simply to an increase in viscosity.

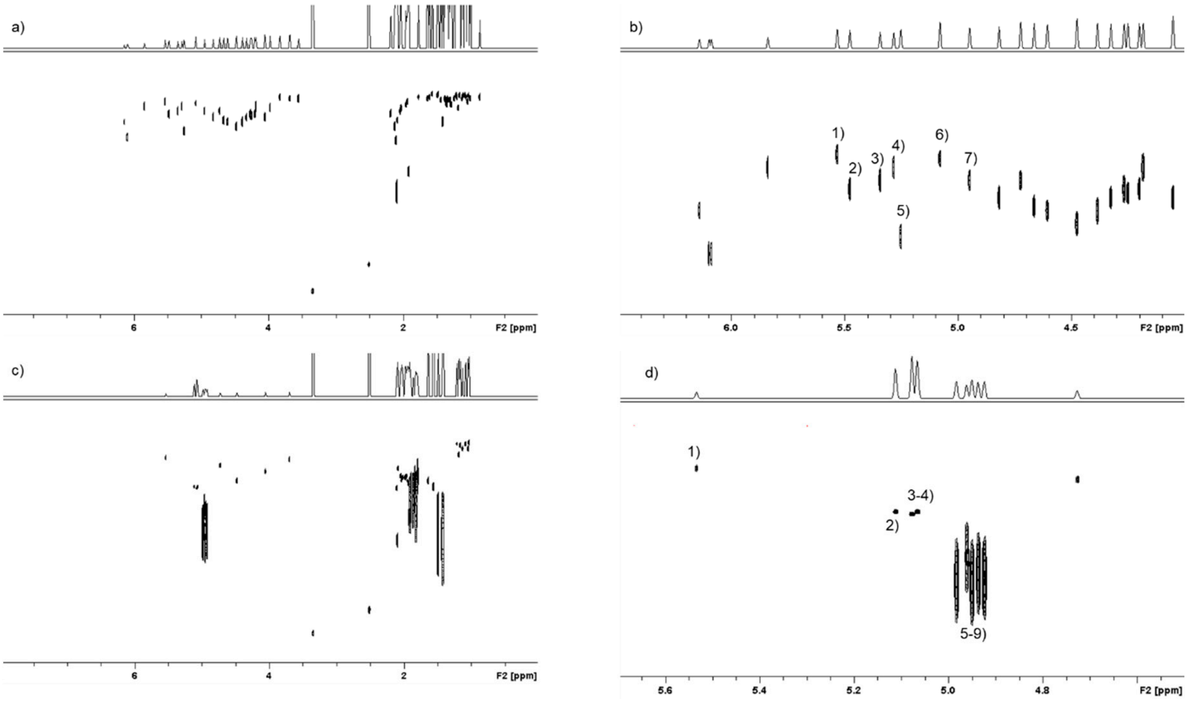

Figure S6. DOSY-NMR for a) 7days 285nm squalene b) with enlargement and c) 7 days 285nm squalene plus squalene spike s) with enlargement

Table S5 Diffusion coefficient for squalene and squalene+ squalene Spike irraditated at 285nm over 7 day. *prefixed numbers are the signals indicated in Figure S6.

Sample	Chemical Shift signals	Diff.Coeff (*10^{-10}) m/s^2	Predicted MW
**squalene**	1)5.53ppm*	1.50	1145
	2)5.48ppm*	1.67	899
	3)5.34ppm*	1.63	949
	4)5.28ppm*	1.57	1032
	5)5.25ppm*	1.95	638
	6)5.08ppm*	1.52	1111
	7)4.94ppm	1.63	949
**squalene + squalene Spike (added after 7 days)**	1)5.53ppm*	1.62	962
	2)5.11ppm*	2.10	543
	3)5.08ppm*	2.11	538
	4)5.06ppm*	2.10	543
	5)4.98ppm*	3.11	240
	6)4.96ppm*	2.75	309
	7)4.95ppm*	3.15	235
	8)4.93ppm*	3.01	257
	9)4.92ppm*	3.07	247

S6 Extended time squalene control under ambient light

A squalene sample was exposed to ambient light over a period of 54 days. The NMR spectra (Figure S7) indicate the sample remains largely unchanged, however within the range of 5-11 days new species seems to arise. These could potentially be hydroperoxide species and appear to be in equilibrium with squalene as the squalene signals seem to decrease and then increase in concentration.

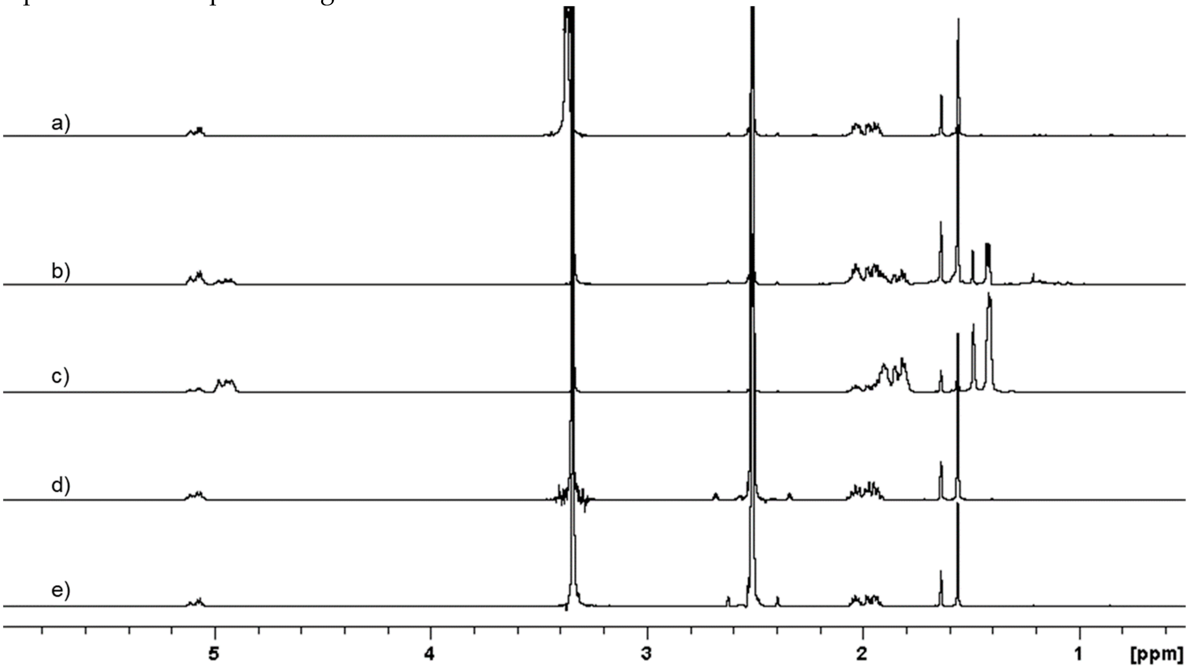

Figure S7. ^{1}H-NMR comparison of squalenesamples left in ambient light for 54d a), 13d b), 11d c) 5d d) and a commercially available sample e).

S7 UV-Vis spectra of Squalene, AIBN & benzoylperoxide in diethyl ether

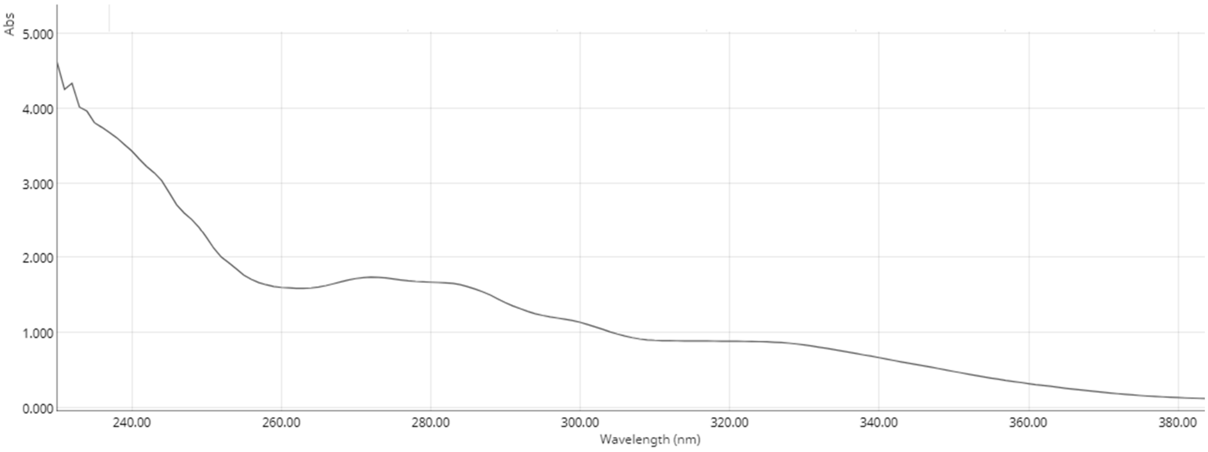

Figure S8. UV-vis spectrum of 10 mg/L squalene in diethyl ether

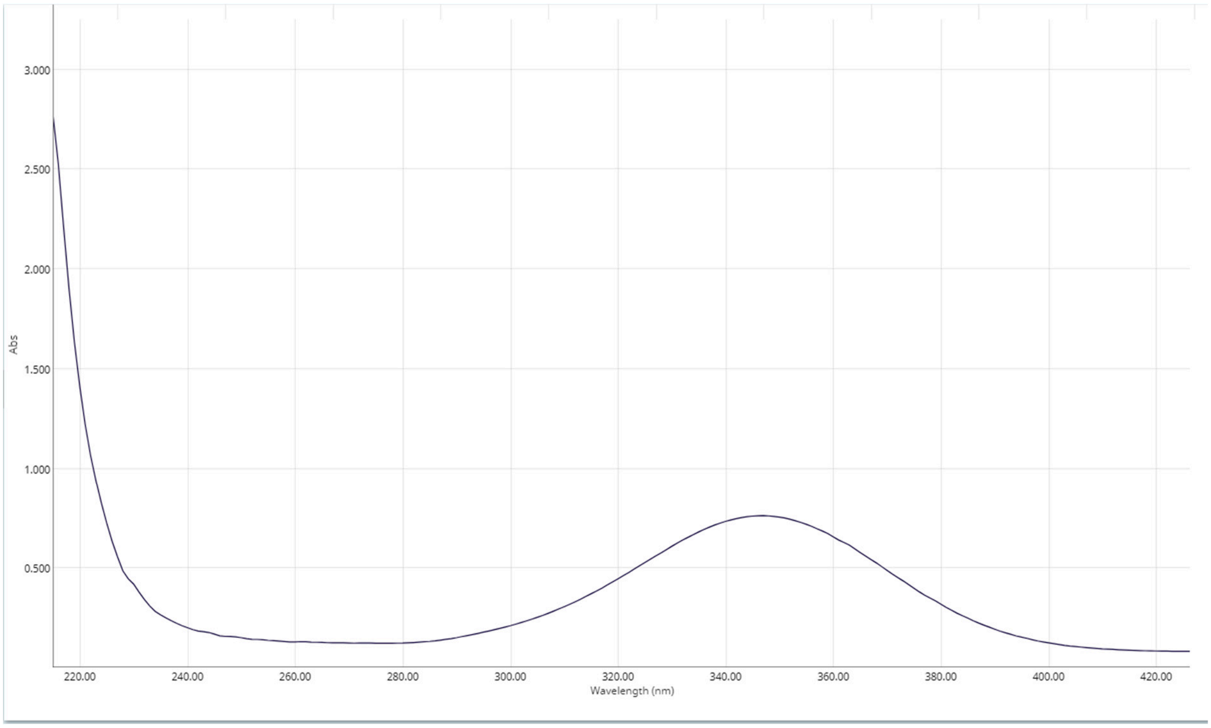

Figure S9. UV-vis spectrum of 10 mg/L AIBN in diethyl ether

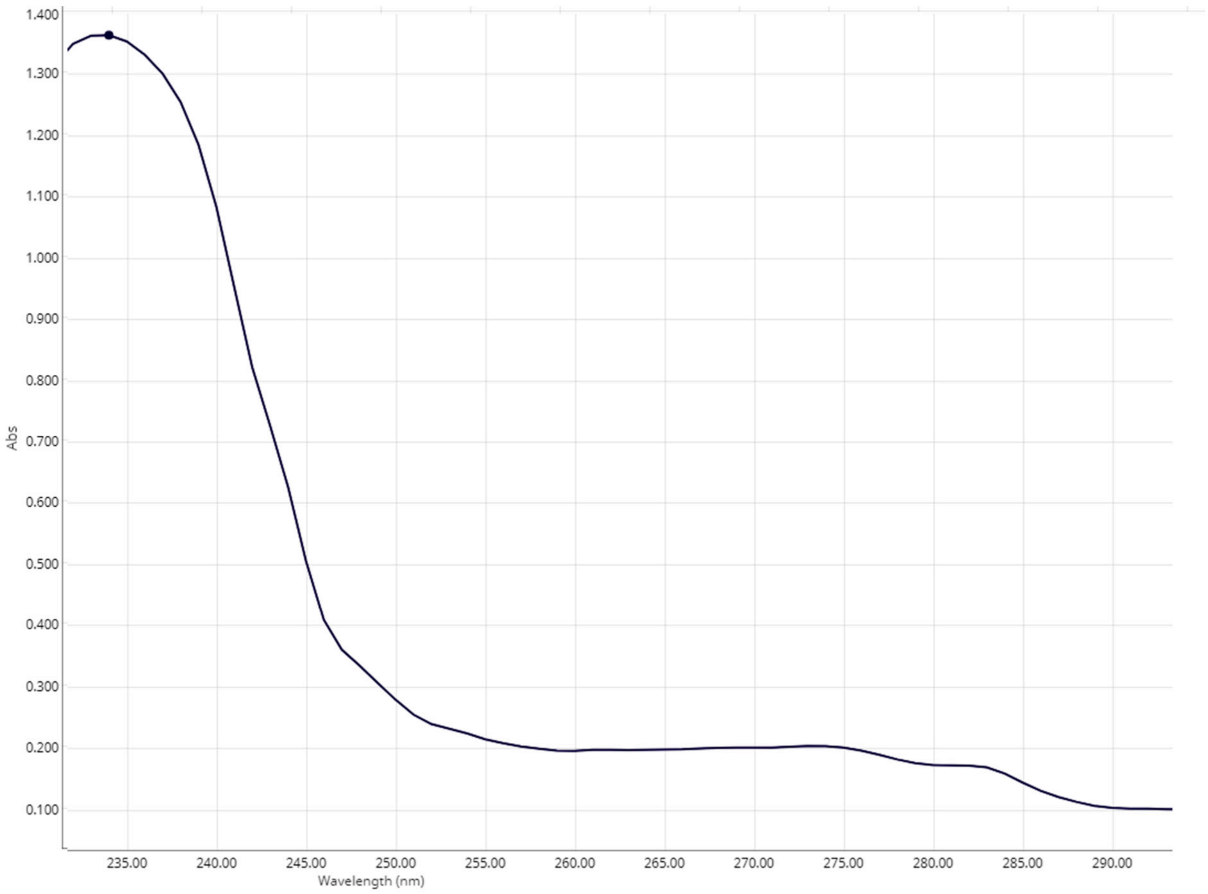

Figure S10. UV-vis spectrum of 1mg/L benzoylperoxide in diethyl ether
